# Supplementary material for: Behavioural and neurogenetic evidence for emotion primitives in the fruit fly Drosophila from an open field test
Source: eLife. 2026 Nov 2;14:RP107110. doi: 10.7554/eLife.107110 (PMC13727074; doi:10.7554/eLife.107110)
Supplement: Supplementary file 4 [file elife-107110-supp1.docx]

**Suppl. File 1: Statistics for Figure 5A-B.** The data for the different ZT times were analysed by a Kruskal-Wallis test. If significant differences were found, Dunn’s multiple comparison test was used for pairwise comparison between ZT times.

|  | **Oregon R** | | | **CS** | | | **CS^110^** | | | **Munich** | | | **Hubland** | | | **Finland** | | |
| --- | --- | --- | --- | --- | --- | --- | --- | --- | --- | --- | --- | --- | --- | --- | --- | --- | --- | --- |
| **WAFO (mm)** | mean | sd | n | mean | sd | n | mean | sd | n | mean | sd | n | mean | sd | n | mean | sd | n |
| ZT0 | 2.61 | 0.71 | 23 | 2.60 | 0.54 | 22 | 3.25 | 0.50 | 24 | 3.30 | 0.38 | 21 | 3.37 | 0.48 | 22 | 3.62 | 0.49 | 24 |
| ZT6 | 2.33 | 0.53 | 22 | 2.64 | 0.56 | 22 | 3.26 | 0.34 | 21 | 3.24 | 0.31 | 22 | 3.49 | 0.31 | 24 | 3.51 | 0.21 | 23 |
| ZT12 | 2.68 | 0.79 | 23 | 2.48 | 0.55 | 22 | 3.57 | 0.53 | 24 | 3.21 | 0.26 | 23 | 3.61 | 0.26 | 22 | 3.80 | 0.41 | 24 |
| ZT18 | 2.49 | 0.86 | 22 | 2.70 | 0.68 | 24 | 3.60 | 0.62 | 24 | 3.45 | 0.38 | 23 | 3.39 | 0.45 | 22 | 3.60 | 0.38 | 23 |
| significance | ns | | | ns | | | ZT6 vs.18: p=0.045 | | | ns | | | ns | | | ns | | |
| **TOWA (m)** | mean | sd | n | mean | sd | n | mean | sd | n | mean | sd | n | mean | sd | n | mean | sd | n |
| ZT0 | 0.17 | 0.28 | 23 | 0.39 | 0.33 | 22 | 1.03 | 0.53 | 24 | 0.91 | 0.57 | 21 | 1.43 | 0.73 | 22 | 1.10 | 0.63 | 24 |
| ZT6 | 0.13 | 0.06 | 22 | 0.52 | 0.35 | 22 | 0.97 | 0.41 | 21 | 1.20 | 0.54 | 22 | 1.71 | 0.59 | 24 | 2.07 | 0.61 | 23 |
| ZT12 | 0.16 | 0.16 | 23 | 0.39 | 0.37 | 22 | 0.76 | 0.34 | 24 | 1.19 | 0.70 | 23 | 1.79 | 0.54 | 22 | 1.24 | 0.61 | 24 |
| ZT18 | 0.17 | 0.31 | 22 | 0.50 | 0.39 | 24 | 0.66 | 0.47 | 24 | 1.05 | 0.53 | 23 | 1.34 | 0.62 | 22 | 1.55 | 0.70 | 23 |
| significance | ns | | | ns | | | ZT0vs.ZT18: p=0.024 | | | ns | | | ns | | | ZT0vsZT6: p<0.0001  ZT0vs.ZT6: p=0.002  ZT0vs.ZT18: p=0.037 | | |
