# Supplementary material for: Behavioural and neurogenetic evidence for emotion primitives in the fruit fly Drosophila from an open field test
Source: eLife. 2026 Nov 2;14:RP107110. doi: 10.7554/eLife.107110 (PMC13727074; doi:10.7554/eLife.107110)
Supplement: Peer review history

# Editor report: eLife Assessment

**DOI:** 10.7554/eLife.107110.3.sa0

**Authors:** Hiromu Tanimoto

**Affiliations:** Tohoku University Japan

**Keywords:** Solid, Useful

This **useful** study reports findings that support the use of the Open Field Test in *Drosophila* as a model to study "emotion-like states", which are behavioral responses to several stressful or aversive treatments, and resilience upon their subsequent removal. Behavioral data, by employing established stress-causing treatments and genetic manipulations, are **solid**. The main advance of this work over previous *Drosophila* work using a similar experimental setup is systematic analysis of various stressors.

# Referee report: Reviewer #1 (Public review)

**DOI:** 10.7554/eLife.107110.3.sa1

Summary:

Animal behavior is continuously influenced by the internal state moment by moment, including emotion primitives as the authors pointed out. Although emotion is a more human-related state, evolutional conservation is undeniable, which can be inferred by the behavioral manifestation. To further elaborate the neuronal mechanisms of emotion primitives, the simplest behavioral parameter related to emotional primitives should be well characterized. In this study, the authors described in detail of wall-following behavior (WAFO) and the total walking distance (TOWA) using flies after subjecting them to various conditions or flies being genetically manipulated according to the previous reports that could affect emotion primitives. Overall, the study is well designed and structured. In addition, the discussion on emotion primitives will be of value to the field.

Strengths:

The strength of this study is its use of a simple behavioral parameter, TOWA, and also a simple design of behavior, WAFO. The importance of the behavioral assay is reproducibility and comparability. In fact, the author demonstrated a summary of comparisons where different treatments result in scalable behavioral changes in WAFO and TOWA.

Conceptual concerns:

My suggestion to strengthen the authors' conclusion that "TOWA can be interpreted as a behavioral proxy for exogenously induced arousal" was to show that an increase in TOWA after stress exposure can be observed in a small (1-cm) arena that acts as an exogenous arousing stimulus, but not in a larger arena (>6.6 cm) where such arousing effects are absent. This comparison would demonstrate that basal locomotor activity measured in larger arenas is not altered by stress, whereas the additional component observed in smaller arenas reflects stress-induced internal state. Therefore, the authors would be able to distinguish clearly the effects of stressors or experiences on either simple locomotion or an emotion-like internal state. Then the future works can follow this protocol using smaller and larger arena to assess emotion-like internal state.

I appreciate the significant authors' efforts to monitor TOWA using arenas with different diameters up to 6 cm. However, the conclusion was unfortunately the same as that obtained using the 1-cm arena. As the authors commented, flies do not show persistent and quantifiable wall-following in arenas larger than 5.8 cm, which limits further examination of this question. I personally agree with the authors' interpretation, but I hope that the authors obtain more definitive experimental contrasts to support this claim in the future study.

# Referee report: Reviewer #2 (Public review)

**DOI:** 10.7554/eLife.107110.3.sa2

Summary:

In terms of data, the revised manuscript is by and large the same, though the authors added new experiments examining the effects of the Open Field Test (OFT) arena size (Fig. 1 Supplement 1) and sex and mating status (Fig. 6). The authors have also provided textual revisions, partially addressing my previous major criticism about novelty over the work of Mohammad et al., 2016, Curr Biol. They argue that the main advance is the systematic inclusion of Total Walking (TOWA) data (e.g. Introduction, page 6 top in the tracked changes document). While I am still not convinced that the findings represent a huge leap forward over that previous work, the authors' systematic analysis is very nice and may prove of use to those seeking to develop *Drosophila* as a model for studying emotion primitives.

Strengths:

The main strength of the paper is the rigorous use of several stressful or aversive treatments and their subsequent removal to show that WAFO is a robust proxy for stress-like emotional primitives across multiple stimuli. The pharmacological, molecular, and neuronal activity manipulations, although more limited in scope, lend further credence to the authors' central claim.

Weaknesses:

The authors have addressed some of my previous points with textual revisions and in their rebuttal. As stated above, the conceptual advance over Mohammad et al. remains in my opinion limited, but I appreciate that this point is now clearly discussed in the manuscript.

# Author comment: Author response

**DOI:** 10.7554/eLife.107110.3.sa3

**Authors:** Yi Lueningschroer-Wang, Emilia Derksen, Hannah Wilde, Maria Steigmeier, Christian Wegener

**Affiliations:** Julius-Maximilians-Universität Würzburg, Biocenter, Theodor-Boveri-Institute, Neurobiology and Genetics Würzburg Germany; Julius-Maximilians-Universität Würzburg, Biocenter, Theodor-Boveri-Institute, Neurobiology and Genetics Würzburg Germany; Julius-Maximilians-Universität Würzburg, Biocenter, Theodor-Boveri-Institute, Neurobiology and Genetics Würzburg Germany; Julius-Maximilians-Universität Würzburg, Biocenter, Theodor-Boveri-Institute, Neurobiology and Genetics Würzburg Germany; Julius-Maximilians-Universität Würzburg, Biocenter, Theodor-Boveri-Institute, Neurobiology and Genetics Würzburg Germany

The following is the authors’ response to the original reviews.

**Public Reviews:**

**Reviewer #1 (Public review):**

Strengths:

The strength of this study is its use of a simple behavioral parameter, TOWA, and also a simple design of behavior, WAFO. The importance of the behavioral assay is reproducibility and comparability. In fact, the author demonstrated a summary of comparisons where different treatments result in scalable behavioral changes in WAFO and TOWA.

We appreciate this assessment and fully agree that the simplicity of the assay and the demonstration of its scalability and reproducibility are a strength.

Weaknesses:

The weakness of the study is the lack of further experiments to support their assumption related to TOWA. The authors suggested that TOWA can be interpreted as a behavioral proxy for exogenously induced arousal. However, it could be interpreted as higher activity, although the authors argued that the circadian clock increasing locomotor activity around ZT0 and ZT12 does not affect TOWA, and therefore TOWA is not related to the locomotor activity per se. As the author cited, flies lose locomotor activity in the circular arena of 6.6 cm in diameter, whereas they continuously move during a 1-h recording in the authors' arena of 1 cm in diameter.

I would agree that the arena of 1 cm in diameter, but not 6.6 cm in diameter, serves as an exogenous stimulus inducing arousal, and TOWA is manifested by arousal. However, TOWA would also be affected by other behavioral parameters, including the activity, motivation for exploration, or perception of the space. Therefore, it could be reasonable to re-examine some of the flies tested in this study in the circular arena of 6.6 cm in diameter. If arousal is biased by the components presented in Figure 6 and TOWA can assess mainly exogenously induced arousal, the treatment altering TOWA in the arena of 1 cm in diameter would not affect their behavior in the arena of 6.6 cm in diameter. My concern is that Figure 6 may demonstrate too simplistic a diagram to interpret the results. I would suggest adding the experiments using the arena of 6.6 cm diameter or softening the argument.

We are grateful that you prompted us to investigate the relation between TOWA and arousal and different arena diameters in more depth. Based on your comments, we compared naïve and stressed behaviour between arena diameters of 1, 2.2 and 5.8 cm. The sizes were chosen as to optimally comply with the camera field of view in our setup. Naïve flies showed stable locomotor activity throughout the 60 min of recordings in the different arenas (new Figure 1 – figure supplement 1 A-B’’). Moreover, no significant difference in TOWA over the first 10 min was found between the different arena sizes (new Figure 1 – figure supplement 1 C). This suggests to us that arenas with a diameter smaller than 6.0 cm (and not only with a 1 cm diameter) induce some form of activity that resembles stimulated activity as defined by Meehan and Wilson (1987). A mechanical shock (shake) resulted in significantly increased WAFO in all arena sizes (new Figure 1 – figure supplement 1 D-D’’). We did not test the effects in larger arenas > 5.8 cm, as we found that flies do not longer show persistent and quantifiable wall following. We started to see that also in few flies in the 5.8 cm arena – these few flies were excluded from our analysis presented in new Figure 1 – figure supplement 1. Unlike the naïve response, the stress-induced response in WAFO and TOWA appears to be transient (new Figure 1 – figure supplement 2), which is in line with the definition of emotions as a transient state.

**Reviewer #2 (Public review):**

Summary:

Strengths:

The main strength of the paper is the rigorous use of several stressful or aversive treatments and their subsequent removal to show that WAFO is a robust proxy for stresslike emotional primitives across multiple stimuli. The pharmacological, molecular, and neuronal activity manipulations, although more limited in scope, lend further credence to the authors' central claim.

We are glad about this assessment and share your opinion.

Weaknesses:

The conceptual advance of this research is unclear, as previous work (Mohammad et al., 2016, Curr Biol.) carried out similar treatments and manipulations and reached largely similar conclusions.

Thank you very much for bringing this up. We rewrote respective parts of the introduction (second last paragraph) and discussion to more clearly outline the advances over the previous work by Mohammad et al. 2016. While our study builds upon Mohammad et al. 2016, the conceptual advance and novelty is that we constitute and treat TOWA as a second and independent dimension equal to WAFO in the OFT. Mohammad et al. had measured locomotor activity (reported as average speed in their paper (total distance walked/time of recording)), but primarily to test the dependency of WAFO on locomotor activity. They found that WAFO metrics were poorly correlated with average walking speed, showing a significant degree of independence of both measures – a finding that our results confirm. However, unlike us, they did not consider average speed/TOWA further for their analysis, possibly because they focused on anxiety-like behaviour while our study looked broader on emotion-like behaviour in general. We further used a round (not square) arena to exclude “cornering” in order to reduce the complexity of the assay, which may explain differences of observed speed/TOWA between our studies.

Moreover, while WAFO is a good proxy for 'stress', I am not convinced that TOWA necessarily represents an emotional state in all cases. Indeed, as the authors themselves acknowledge, changes in total walking may be associated with other factors, such as starvation-induced hyperactivity, physical exhaustion after sleep deprivation, increased sex drive after mating, alcohol sedation, etc.

Your comment raises a question in comparative research on emotions which is very difficult if not impossible to conclusively answer. At first sight, the most conservative stance seems to be to completely disregard the idea of emotions and affective experiences in animals. This, however, would mean that we cannot use animal models to study the basics of emotions (=emotion primitives) and would ignore that by all likelihood emotions are a product of evolution and hence should exist at least in more basic forms in animals. Obviously, we have no means to ask flies or any other animal whether they connect “hunger” or “mating” to a feeling or an emotional state (which must not be conscious) but can only observe the behaviour. We here adopt the often-cited “Pankseppian” view (based on the book of Jaak Panksepp: “Affective Neuroscience”) and firmly believe that – in order to fully understand how the brain drives behaviour- we also need to take affective states into account that bias behaviour towards adaptive responses.

In short, we are unfortunately unable to give a clear and definite answer to your comment whether TOWA represents an emotional state in all cases. Perhaps you are right. We believe, however, that a “Pankseppian” view is adequate, and we may ask what evidence exists that shows that starvation-induced hyperactivity or post-mating is not associated with an affective emotion-like state in the fly or any other animal.

Another unclear point is the interpretation of some unexpected results, such as the finding that both serotonin transporter overexpression and its knockdown give the same phenotype.

Thank you very much for this comment, which we also received by reviewer #1. As suggested by the other reviewer, a reasonable mechanism that may underly the similarity of effect after knockdown or overexpression of SerT may be a differential effect on the different serotonin receptor subtypes expressed in the brain. The possibility that the concentration-dependent effect of a biogenic amine follows a U-shape is further reasonable and has been demonstrated for dopamine. For example, the relationship between cognitive performance or working memory and dopamine levels in primates follows an inverted U-shape (see e.g. Cools and D’Esposito 2011 10.1016/j.biopsych.2011.03.028, Desimone 1995 10.1038/376549a0). Also in *Drosophila*, both reduced and increased dopamine levels lead to increased male-to-male courtship behaviour (Liu et al. 2008 10.1523/JNEUROSCI.5290-07.2008, Liu et al. 2009 10.1371/journal.pone.0004574). While we are unaware of similar examples for serotonin, we note that serotonin levels must be kept at optimum level during development – both higher and lower than optimum levels result in behavioural impairments in adults (see e.g., Shah et al. 2018 10.3389/fnbeh.2018.00114). We have now extended the discussion accordingly.

Finally, there are some issues with the use of the OFT in rodent research (e.g., inconsistent effects of anxiolytic drugs; see Rosso et al., 2022, Neurosci Biobehav Rev., for a meta-analysis). These should be explained to place the *Drosophila* findings in their appropriate context.

Thank you very much for bringing this systematic review to our attention which assessed the usefulness of various behavioural tests including the OFT to study the effect of anxiolytic drugs in rodents. Overall, the review casts “serious doubt on both construct and predictive validity” of behavioural tests for anxiolytics. While diazepam (the only drug used in our study) was the drug with the most consistent effects across the analysed behavioural assays, only 59% of the OFTs revealed significant effects. We were already aware of earlier findings in the same direction (Prut and Belzung 2003 10.1016/s0014-2999(03)01272-x), but as we only used one drug did not include a discussion in the manuscript. Unfortunately, the number of studies employing the OFT in flies is very small and does not yet allow for a similar comparison. We now changed the respective sentences in the discussion:

“In rodents, diazepam mostly but not consistently leads to an anxiolytic response in the OFT behaviour which questions the usefulness of the OFT for testing anxiolytic drugs (see (Prut and Belzung 2003; Rosso et al. 2022)).”

**Recommendations for the authors:**

**Reviewer #1 (Recommendations for the authors):**

Overexpression of SerT suppressed increased WAFO after electric shocks. However, knockdown of SerT did similar. It is worth reporting this finding, but possible mechanisms could be mentioned in the manuscript. For example, given that flies carry five serotonin receptor genes, upregulation of overall serotonin level may affect the specific serotonin receptor, but downregulation of it may affect other receptors, leading to unexpected outcomes. Exploring any other possibilities could be better to add to guide future research.

Thank you very much for this comment and the suggestion of a reasonable mechanism that may underly the similarity of effect after knockdown or overexpression of SerT. The possibility that the concentration-dependent effect of a biogenic amine follows a U-shape is further reasonable and has been demonstrated for dopamine. For example, the relationship between cognitive performance or working memory and dopamine levels in primates follows an inverted U-shape (see e.g. Cools and D’Esposito 2011 10.1016/j.biopsych.2011.03.028, Desimone 1995 10.1038/376549a0). Also in *Drosophila*, both reduced and increased dopamine levels lead to increased male-to-male courtship behaviour (Liu et al. 2008 10.1523/JNEUROSCI.5290-07.2008, Liu et al. 2009 10.1371/journal.pone.0004574). While we are unaware of similar examples for serotonin, we note that serotonin levels must be kept at optimum level during development – higher or lower levels result in behavioural impairments in adults (see e.g. Shah et al. 2018 10.3389/fnbeh.2018.00114). We have now extended the discussion accordingly.

**Reviewer #2 (Recommendations for the authors):**

(1) The advance over Mohammad et al., 2016, Curr Biol. must be clearly and emphatically articulated in the Introduction and/or Discussion. It is otherwise impossible to appreciate what is conceptually novel about this work.

We have now rewritten parts of the two last paragraphs in the introduction to make the advances clearer. As outlined above, we conceptually advanced the analysis of OFT behaviour by integrating TOWA as a second and independent dimension in our analysis. During the revision process, we have spent great effort to better characterise the nature of the locomotor activity encountered in the OFT (Figure 1 – supplementary figures 1 and 2). Also, this is an advancement over previous studies, including Mohammad et al. 2016. We further included new results on the general effect of neuropeptides (*silver* mutants, impaired in neuropeptide processing).

(2) The most important metric for a stress-like emotional primitive is WAFO. Therefore, figures should be revised in a way that highlights WAFO differences. Figure 4 is a good example of this. In contrast, in Figures 1-3, the WAFO box-and-whisker plots are very small and obscured under the raw WAFO-TOWA plots, which are difficult to see (especially given the light blue background of all plots) and redundant. I strongly recommend just showing the WAFO box-and-whisker plots for the sake of visibility, clarity, and brevity.

Although we understand your reasoning, we would like to stick with the old figures as we consider TOWA as important to characterise the OFT response as WAFO (see our comments above regarding the conceptual advances of our study over Mohammad et al. 2016). It is true that Figures 1-3 are small, but at least in our print-out well legible. Further, it appears that in the current version of the eLife system the resolution is downsampled. In addition, we anticipate that in the version of record figures can be enlarged online as in other eLife articles.

(3) Given the criticisms against the OFT in rodent research, one of which is inconsistent effects of anxiolytic drugs (Rosso et al., 2022, Neurosci Biobehav Rev.), it may be useful to expand pharmacological treatments beyond diazepam.

As our focus is not on the testing of anxiolytic drugs and since it was already very difficult to be granted access to diazepam (we are not at a medical institution), we refrained from testing further drugs. Moreover, as rightfully mentioned by you, the OFT may not be the best test for the efficacy of anxiolytic drugs. On the other hand, diazepam was the most consistent anxiolytic in the OFT in rodents (see Rosso et al. 2022).

(4) The authors should show results of the effects of at least some stressors/punishments on WAFO/TOWA of female flies to understand if observed effects are sex-specific.

Thank you very much for bringing this topic to our attention. To test whether the effects are sex-specific, we now performed several new experiments. First, we compared the naïve OFT response of mated and unmated males and females (see new Figure 6). This revealed that without prior stress treatment, the WAFO response is independent of sex and mating status. In contrast, the naïve TOWA response turned up to be sex- and mating state-specific (see new Figure 6). To test whether the mated females show a different stress-induced OFT response to males, we applied mechanical stress (shake) that we had also used to assess the effects of arena diameter (Figure 1 – supplementary Fig. 1 D-D’’). After a first round of shaking, females showed increased TOWA, but WAFO was unaffected. A second round of shaking, however, led to a significant increase in WAFO and TOWA. This suggests that the OFT response is qualitatively similar between the sexes and mating status, yet the threshold for elicited responses differs between males and females. We now added a respective paragraph to the main text in the results section plus a new figure (Fig. 6).
